# Flower Development and Sex Determination between Male and Female Flowers in *Vernicia fordii*

**DOI:** 10.3389/fpls.2017.01291

**Published:** 2017-07-20

**Authors:** Yingji Mao, Wenbo Liu, Xue Chen, Yang Xu, Weili Lu, Jinyan Hou, Jun Ni, Yuting Wang, Lifang Wu

**Affiliations:** ^1^Key Laboratory of Ion Beam Bioengineering, Institute of Technical biology and Agriculture Engineering, Hefei Institutes of Physical Science, Chinese Academy of Sciences Hefei, China; ^2^School of Life Science, University of Science and Technology of China Hefei, China; ^3^Biotechnology Center, Anhui Agriculture University Hefei, China; ^4^School of Pharmacy, Anhui Medical University Hefei, China; ^5^The Sericultural Research Institute, Anhui Academy of Agricultural Science Hefei, China

**Keywords:** tung tree, monoecious, reproductive development, staminode, sex determination, transcriptomics

## Abstract

*Vernicia fordii* is a monoecious and diclinous species with male and female flowers on the same inflorescence. Low female to male flower ratio is one of the main reasons for low yield in this species. However, little is known of its floral development and sex determination. Here, according to the results of scanning electron microscopy and histological analysis, the floral development of *V. fordii* was divided into 12 stages and the first morphological divergence between the male and female flowers was found to occur at stage 7. The male flowers are always unisexual, but the female flowers present bisexual characteristics, with sterile stamen (staminode) restricted to pre-meiosis of mother sporogenous cells and cell death occurring at later development stages. To further elucidate the molecular mechanism underling sex determination at the divergence stage for male and female flowers, comparative transcriptome analysis was performed. In total, 56,065 unigenes were generated and 608 genes were differentially expressed between male and female flowers, among which 310 and 298 DEGs (differentially expressed genes) showed high expression levels in males and females, respectively. The transcriptome data showed that the sexual dimorphism of female flowers was affected by jasmonic acid, transcription factors, and some genes related to the floral meristem activity. Ten candidate genes showed consistent expression in the qRT-PCR validation and DEGs data. In this study, we provide developmental characterization and transcriptomic information for better understanding of the development of unisexual flowers and the regulatory networks underlying the mechanism of sex determination in *V. fordii*, which would be helpful in the molecular breeding of *V. fordii* to improve the yield output.

## Introduction

Two lakhs fifty thousand to Three lakhs fifty thousand flowering plants are known in the world, about 90% of which are hermaphroditic, producing male and female sex organs on the same flower, while 10% are completely unisexual (Charlesworth and Guttman, 1999; Paton et al., 2008; Heijmans et al., 2012; Renner, 2014). Approximately half of these unisexual plants are monoecious species with separate male and female flowers on the same plant, such as maize (*Zea mays*), cucumber (*Cucumis sativus*), and melon (*Cucumis melo*). The other half are dioecious with male and female flowers on separate plants, such as white campion (*Silene latifolia*), papaya (*Carica papaya*), hemp (*Cannabis sativa*), and annual mercury (*Mercurialis annua*). In flowering plant, sex determination is a developmental process that facilitates allogamy to support fitness and survival. In unisexual flowers, the process of sex determination is performed by the selective abortion or arrest of either male or female reproductive organs. The mechanisms of sex determination have been investigated in some model plants, such as maize and cucumber, and are mainly affected by genetics, plant hormones and environmental factors (Bai and Xu, 2013; Li and Liu, 2017).

For dioecious species, sex determination genes are often located in non-recombining regions of the sex chromosome (Zhang et al., 2014). In white campion, the Y chromosome is the largest chromosome in the genome and also the primary factor that determines floral sexuality, with at least one region suppressing female expression and another promoting male development (Juarez and Banks, 1998; Zhang et al., 2014). In papaya, sex determination is controlled by two types of Y chromosomes that control the male Y and the hermaphrodite Y^h^ (Ming et al., 2007). Sex determination genes have been identified and investigated in maize and cucurbits to improve the understanding of the regulatory network for sex determination. A sex determination model for melon was recently proposed in which the expression of *CmWIP1*, required for carpel abortion in the male flower, is dependent on the non-expression of *CmACS11*. In this model, the expression of *CmACS*-7, which leads to stamen repression in the female flower, is dependent on the non-expression of *CmWIP1* (Boualem et al., 2008, 2015; Martin et al., 2009). It is known that plant hormones regulate plant growth and development and also affect sex differentiation in some monoecious and dioecious plants. Ethylene is the primary hormone promoting female flower development in melon and cucumber, whereas gibberellins have opposite effects in these plants (Yamasaki et al., 2005). In maize, gibberellins induce feminization while jasmonic acid and brassinosteroids induce masculinization (Yamasaki et al., 2005; Zhang et al., 2014). Environmental factors also affect sex determination in many species, such as high temperature and long-day conditions, which can promote male flowers, whereas low temperature and short-day conditions induce feminization in cucumber (Yamasaki et al., 2005; Li et al., 2012).

For the formation of unisexual flowers, the developmental fate of inappropriate sex organs is determined at different developmental stages in different plants (Diggle et al., 2011). There are four stages of inappropriate sex organ abortion: stage 0 occurs before the initiation of the stamen or carpel primordia; stage 1 is the stage containing the early stamen or carpel primordia; stage 2 represents pre-meiosis; and stage 3, post-meiosis (Diggle et al., 2011). In maize, the formation of unisexual flowers results in the abortion of pistil primordia in the tassel silkelets and the secondary florets of ear spikelets, mediated by programmed cell death (PCD), and the arrest of stamen primordia in ear spikelets, which involves repression of the cell cycle (Calderon-Urrea and Dellaporta, 1999; Kim et al., 2007). In cucumber, the arrest of the stamen in female flowers is mainly restricted to the anther at stage 7 with DNA damage, which can be specifically detected by TUNEL assay. However, the arrest of carpel development in male flowers occurs prior to ovary differentiation without DNA damage and TUNEL signal (Hao et al., 2003; Bai et al., 2004). Moreover, the vestiges of a stamen and carpel are left in the female and male flowers respectively and remain metabolically active (Hao et al., 2003). In contrast, hemp and annual mercury lack any vestiges of inappropriate sex organs and present as completely unisexual (Diggle et al., 2011). Taken together, the transition from bisexual to unisexual features may occur in any cell specification stage of floral development with different processes for each diclinous species.

*Vernicia fordii* (tung tree) is a perennial deciduous woody oil plant in subtropical and tropical areas with tremendous potential as raw material to produce biodiesel (Chang et al., 2013). The seeds of the tung tree contain 50–60% oil with approximately 75% α-eleostearic acid (9 *cis*, 11 *trans*, and 13 *trans* octadecatrienoic acid), which determines the quality of tung oil (Weichang et al., 2008). In recent years, the tung tree has been highlighted as an important woody bioenergy plant in China for resolving the increasing energy crisis. Tung trees are monoecious and predominantly synoecious plants, the flowers of which are developed in panicled cymose inflorescences that are terminal and solitary on new branches. The number of staminate flowers greatly exceeds that of the pistillate flowers and the average ratio of female to male flowers is 1:27, which results in low yield (McCann, 1942). Therefore, it is important to investigate the flower development and sex determination processes in the tung tree for further alteration of the sex ratio and to increase in the yield. However, few studies are available on floral development in the tung tree (Abbott, 1929; McCann, 1942). The mechanisms underlying the transition from a bisexual to a unisexual flower and floral organ development are clear in some species, such as *Phoenix dactylifera* (Daher et al., 2010), *Vitis vinifera* (Caporali et al., 2003), *S. latifolia* (Farbos et al., 1997), and *Xanthoceras sorbifolia* (Zhou et al., 2012), but unclear in the tung tree.

In this paper, we report a detailed morphogenetic and transcriptomic analysis of floral development in *V. fordii*. The male flowers are always unisexual, but the female flowers present bisexual characteristics with sterile stamen (staminode) restricted to the anther during the early development stages, and while cell death occurs in later development stages. Comparative transcriptome sequencing of the male and female flowers was carried out to better understand the molecular regulatory mechanism governing unisexual flower development in *V. fordii*.

## Materials and methods

### Plant materials

Six hundred tung trees used in this experiment were an Anhui local cultivar “Duisuitong,” the seeds of which were sown and cultivated in the test field of the Hefei Institutes of physical science, Chinese Academy of Sciences, Anhui, China, at 2011. The terminal buds were randomly collected every week at various growth stages, from April 2015 to April 2016. However, the samples were collected every 3–4 days at the beginning of the reproductive phase in July 2015 and every 2 weeks during the phase of reproductive dormancy from November 2015 to January 2016. All of the sampled buds were dissected by removing their scales and leaves and fixed in FAA (70% ethanol: acetic acid: formaldehyde, 90:5:5, v/v) or 5% glutaraldehyde at 4°C for further histological and SEM studies, respectively.

### Histological analysis

All samples in FAA were vacuum filtered for ~30 min. Then the samples were dehydrated by sequentially immersing through graded ethanol: 50, 70, 85, 90, and 100% ethanol, each for 2 h at room temperature. Then, the samples were incubated overnight into the fresh 100% ethanol containing 0.1% Eosine that makes the samples more visible during embedding and sectioning. The following day, the dehydrated materials were incubated in the ethanol gradually with xylene as follows: 100% ethanol for 1 h, ethanol/xylene (3:1, v/v) for 1 h, ethanol/xylene (1:1, v/v) for 1 h, ethanol/xylene (1:3, v/v) for 1 h, two changes of xylene for 1 h. At the end of the day, the materials were set in xylene/paraffin (1:1, v/v) overnight at 60°C oven. The third day, the tissues were embedded by regularly adding more paraffin (every hour) until the pure molten wax at the end day for incubation 60°C overnight. The final day, the paraffin was replaced three times at intervals of 3 h and the tissues were infiltrated by vacuum 30 min at 60°C. Samples were longitudinally and transversely sectioned into pieces with 8 um thickness. Sections were double stained with Safranin O and Fast Green FCF according to the Sass's method (Ruzin, 1999). The sections were deparaffinized 15 min in xylene two times and washed in water. Then, the sections were stained for 12 h in aqueous Safranin O (1% w/v) and rinsed in water until no more dye. After that, sections were dehydrated for 2 min each through the following ethanol series: 30, 50, 70, and 95% ethanol. The sections were counterstained 30 s in 95% ethanol plus Fast Green FCF (0.1% w/v) and washed 2 min in 100% ethanol two times. Subsequently, the slides were dipped 5 s in a mixture of methyl salicylate and xylene (1:1, v/v) and cleared in 100% xylene for two changes. Finally, the sections were mounted and covered with glass coverslip. The sections were observed and photographed under the SZX10 stereo microscope (Olympus, Japan) and the BX53microscope (Olympus, Japan).

### Scanning electron microscopy

The fixed and dissected materials were dehydrated by passing them through ethanol gradually with an acetone series: 30, 50, 70, 80, 90, and 100% ethanol for 15 min each, ethanol/acetone (1:1, v/v) for 30 min and 100% acetone for 30 min. The materials were critical-point dried using liquid CO_2_ (Emitech K850, UK). All materials were sputter-coated with gold (Hitachi E-1010, Japan) and examined with an S4800 scanning electron microscope running at an accelerating voltage of 3 kV (Hitachi, Japan). The heights of the floral sex organs (*n* = 10) were measured using ImageJ software (https://imagej.nih.gov/ij/). Data were analyzed statistically using the Origin 8.5 (OriginLab Corporation, USA).

### DAPI staining

Nuclear loss assays were performed using 4′,6-diamidine-2-phenylindole dihydrochloride (DAPI, Sigma). Sections were rehydrated through a graded ethanol series: 100, 95, and 70% ethanol for 5 min each, 0.75% NaCl in 50% ethanol for 2 min and 0.75% NaCl in 30% ethanol for 2 min. Afterward, sections were immersed in 0.75% NaCl for 5 min and washed in PBS solution containing 0.3% Trixton for 5 min. Then, sections were incubated in a 0.3 mM solution of DAPI in PBS for 15 min followed by three washes in PBS and mounting in VECTASHIELD (Vector, USA). The samples were observed and photographed using a BX53 microscope (Olympus, Japan).

### TUNEL assay

The TUNEL (TdT-mediated dUTP nick-end labeling) assay was used to determine whether PCD was occurring in the aborted stamens (staminodes) from *V. fordii* female flowers. We used the DeadEnd Fluorometric TUNEL system according to the manufacturer's instructions (Promega, USA). Sections were counterstained in 1 mg/ml propidium iodide (PI) in PBS and mounted in VECTASHIEDLD (Vector, USA). Slides were examined and photographed under a BX53 microscope using blue and green excitation to visualize the green TUNEL fluorescence and the red PI fluorescence, respectively.

### RNA extraction and sequencing

Male and female flower buds at developmental stage 7 were identified under the SZX10 stereo microscope (Olympus, Japan) and separately collected from the same plant. Three biological replicates were performed for each sample and 30 flowers counted as one sample. All samples were immediately frozen in liquid nitrogen and stored at −80°C for RNA extraction. Total RNA was isolated using RNAiso plus (Takara, Japan), according to the manufacturer's protocol. RNA sequencing was performed by Illumina Hiseq 2500 (Illumina, USA) obtaining reads with 2 × 100 bp length at Shanghai Biotechnology Corporation (Shanghai, China). The paired-end adapter sequences, short sequences (<20 bp) and low-quality reads (Q score <20) were removed. All clean reads were processed with CLC Genomics Workbench software (version 6.0.4) using the scaffolding contig program (word-size = 45, minimum contig length ≥200) and CAP3 EST software to generate *de novo* assembled unigenes (Garg et al., 2011; Su et al., 2011). All sequencing data of male and female flower buds in *V. fordii* are available at the NCBI's Gene Expression Omnibus (accession number: GSE98631).

### Annotation and gene ontology enrichment analysis

To annotate the unigenes, the BlastX program was used to search against the *Arabidopsis thaliana* protein sequence data set (TAIR 10, http://www.arabidopsis.org/) with an *E*-value threshold of 1E-5 (Altschul et al., 1990). Thus, the best-hit *Arabidopsis* homologs [using *Arabidopsis* gene identifiers (AGI)] were mapped to the *V. fordii* unigene ID. Gene Ontology (GO) annotation was performed using the Blast2GO software (Conesa and Gotz, 2008) and functional classification was analyzed with the Classification SuperViewer tool (http://bar.utoronto.ca/; Provart and Zhu, 2003). The unigene sequences were mapped to the KEGG Automatic Annotation Server database to reveal metabolic pathways (Moriya et al., 2007).

The differentially expressed genes (DEGs) between male and female flower buds were determined using the DEGseq program with the RPKM (reads per kilobase per million reads) values and satisfied with the false discovery rat (FDR) values < 0.05 and fold changes ≥2 (Wang et al., 2010).

To further investigate the functions of DEGs, GO enrichment analysis was performed using OmicShare tools, a free online platform for data analysis (http://www.omicshare.com/tools). Firstly, all DEGs were mapped to the GO terms in the Gene Ontology database (http://www.geneontology.org/). Gene numbers were calculated for every term and significantly enriched GO terms in the DEGs compared to the genome background were defined with a hypergeometric test. The calculated *p*-value was subjected to FDR correction, with FDR ≤ 0.05 used as a threshold. GO terms meeting this condition were defined as significantly enriched GO terms in the DEGs. This analysis may be able to recognize the main biological functions of the DEGs. Meanwhile, *V. fordii* DEGs with unique AGIs were submitted to MapMan for categorization of the DEGs based on biological functions (Thimm et al., 2004).

### qRT-PCR analysis

To validate the RNA-seq data, 1 ug RNA was used for cDNA synthesis according to the instructions for the PrimeScript Reverse Transcriptase (Takara, Japan). Tung 60 s ribosome protein L19 (Rpl19b, Genbank accession No. FJ362591) was used as the reference gene (Cao et al., 2013). All of the specific primers for the selected DEGs are shown in Table S1. qRT-PCR was performed using the LightCycler 96 (Roche, USA) and SYBR Green PCR Master Mix. Three biological repeats were applied for each experiment.

## Results

### Phenological growth stages of floral bud

**Figure 1:**
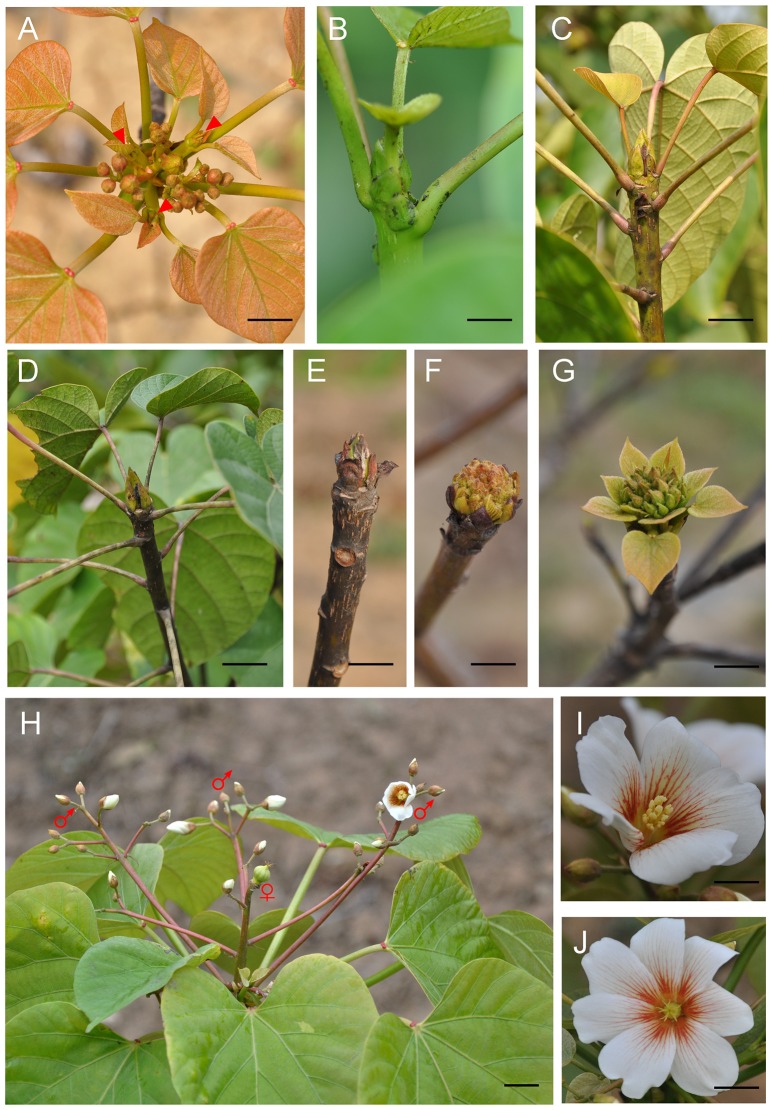
Phenological growth stages and structure of *V. fordii* flower. **(A)** The new shoots (red arrowhead) emerged in April. **(B,C)** The vegetative shoots grew in May and June. **(D)** The inflorescence emerged in July and August. **(E)** The dormancy of floral buds in winter. **(F)** Floral bud breaking and swelling in spring. **(G)** The formation of cymose inflorescence **(H)** Structure of an inflorescence. ♀: male flower, ♂: female flower (petal removed). **(I)** The male flower. **(J)** The female flower. Scale bar = 1 cm.

In April, the inflorescences started to flower after one-year development. At the same time, new shoots were generated from the leaf axil (Figure 1A). The growth of vegetative shoots was robust and vigorous in the next 2 months (Figures 1B,C). The vegetative shoot meristem was transformed into an inflorescence meristem between July and August (Figure 1D). The developmental pattern for the entire inflorescence process was basipetal. The primary inflorescence meristem consecutively differentiated into secondary, tertiary, or quaternary inflorescence, which was subtended by bracts. Meanwhile, the flower primordia started to differentiate. The central and terminal flowers developed first, followed by the lateral flowers. After being subjected to low temperatures in winter, the floral buds were covered by a brown scale for dormancy (Figure 1E). In the spring, the floral differentiation resumed and rapid development took place. Floral buds began to swell and open and the scales loosened and parted (Figure 1F). The rudimentary bracts became visible and increased in length. The main and branch axes lengthened, leading to the formation of a panicled and compact cymose inflorescence (Figure 1G). The first flower opened in April and the flowering period lasted ~3 weeks.

The structure of the inflorescence showed that the rachis may branch dichotomously or trichotomously (Figure 1H). The main and lateral flower stalks are also branched in the same manner. In an inflorescence, female flowers are located at the center of the main or second inflorescence axis, which are surrounded by male flowers. Typically, mature flowers are unisexual. The number of petals varies from five to eight in male flowers (mostly five petals). Eight to twelve stamens are arranged in two whorls that harbor upper and lower layers, respectively (Figure 1I). Female flowers have five to nine petals (mostly eight petals) (Figure 1J), three to five fused carpels with a large ovary, a short style, and the same number of stigmas with two splits.

### Stages of flower development

**Figure 2:**
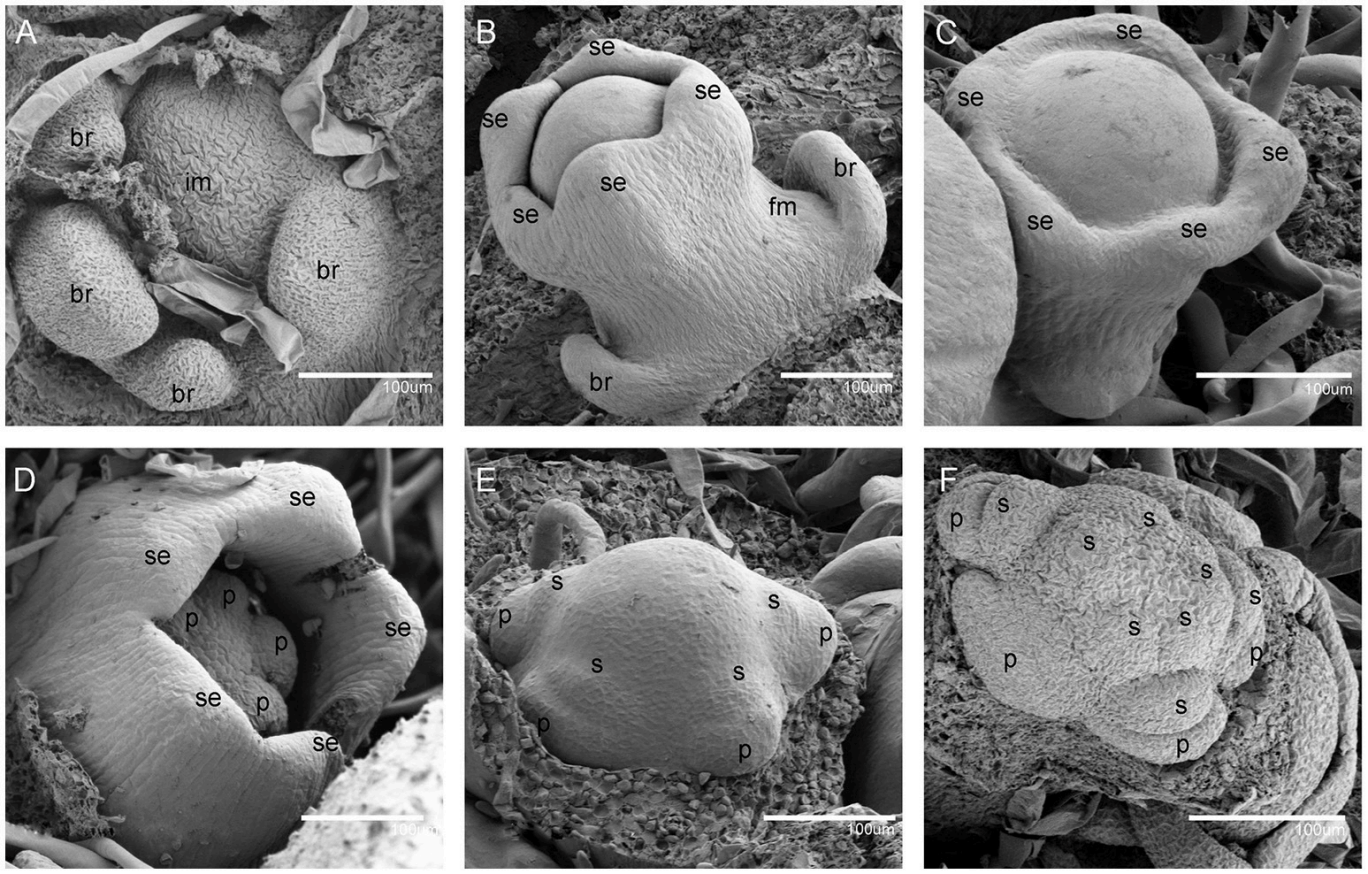
Scanning electron micrographs for *V. fordii* flower in stages 1–6. **(A)** Stage 1: inflorescence meristem was subtended by bracts. **(B)** Stage 2: floral meristem initiated on the flank of inflorescence. **(C)** Stage 3: appearance of sepal primordia. **(D)** Stage 4: formation of petal primordia. **(E)** Stage 5: appearance of lower layer stamens. **(F)** Stage 6: upper layer stamens bulged. im, inflorescence meristem; br, bract; fm, floral meristem; se, sepal; p, petal; s, stamen.

**Figure 3:**
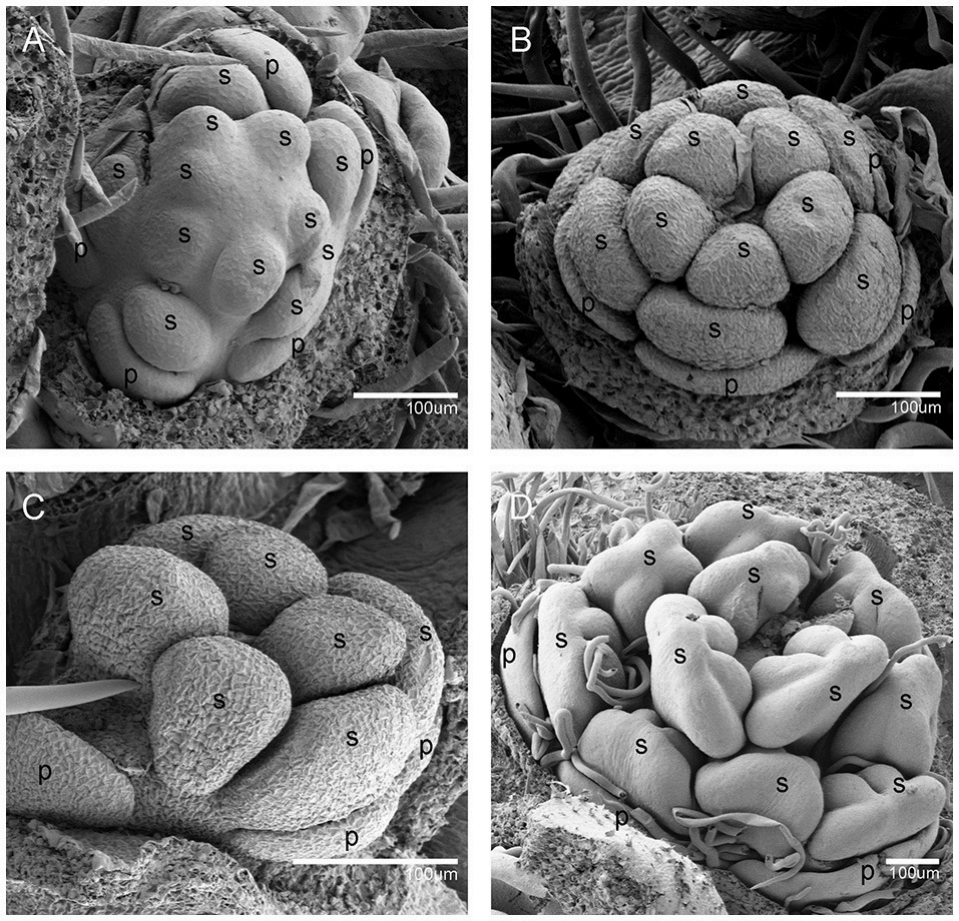
The development of the male *V. fordii* flower during sex differentiation. The sepal primordia have been removed. **(A,B)** Stage 7 in the male flower: the floral meristem was arrested and oval-shaped stamen primordia were formed. **(C)** Stage 8: stamen primordia were transformed from oval-shaped to tongue-shaped and stamens became stalked. **(D)** Stage 9: anthers of male flowers formed convex protrusions on the inner surface. p, petal; s, stamen.

**Figure 4:**
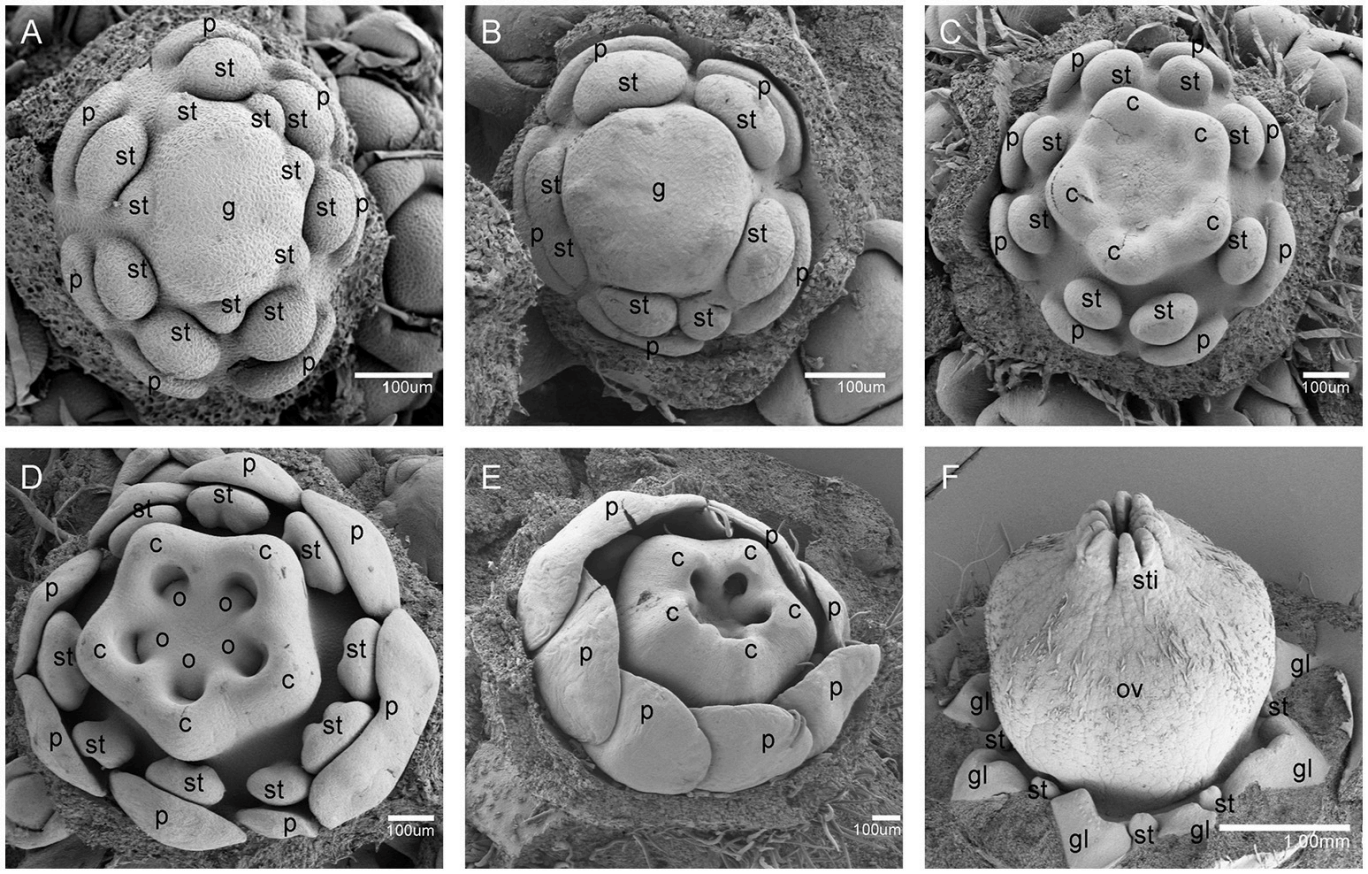
The development of the female *V. fordii* flower during sex differentiation. **(A)** Stage 7 in the female flower: the floral meristem began to bulge and differentiate and the hemispherical gynoecium was established. **(B)** Stage 8: the gynoecium primordium enlarged and formed a flattened and rounded protrusion. **(C)** Stage 9: the carpel primordia appeared at the edge of the gynoecium. **(D)** Stage 10: the ovule primordia emerged. **(E)** Stage 11: the carpels were fused at the base. **(F)** Stage 12: each carpel differentiated into a short style and two-split stigma appeared. p, petal; st, staminode; g, gynoecium; c, carpel; o, ovule; ov, ovary; sti, stigma; gl, gland.

**Figure 5:**
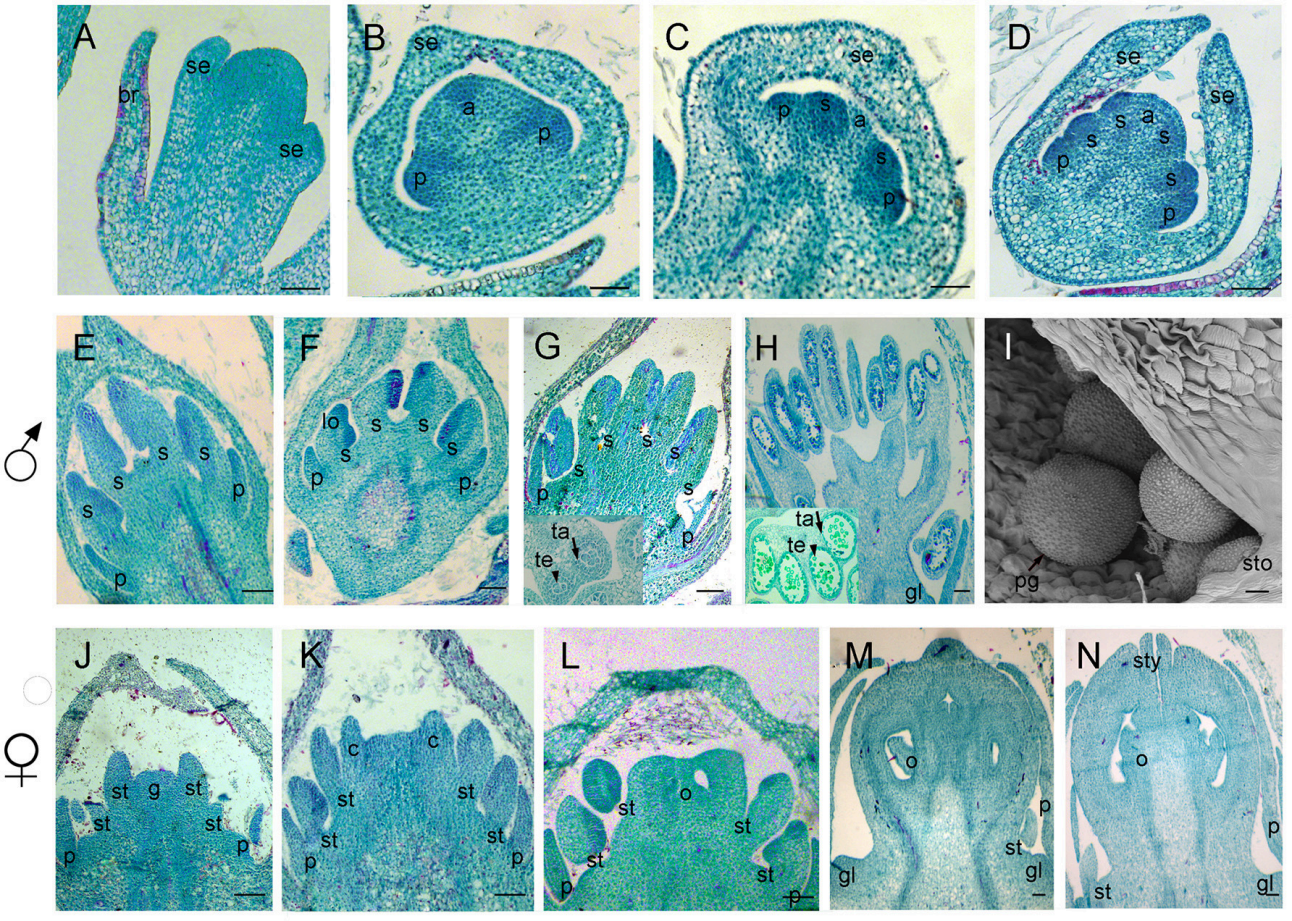
Samples were sectioned into pieces with 8 um thickness. Sections of flower development in *V. fordii*. **(A)** Stage 3. **(B)** Stage 4. **(C)** Stage 5. **(D)** Stage 6. **(E)** Stage 8 in the male flower: the filament and anther appeared. **(F)** Stage 9 in the male flower: locules formed with dense staining. **(G)** Stage 10 in the male flower: meiosis occurred and tetrads were surrounded by tapetum at the inset of the transverse section. **(H)** Stage 11 in the male flower: the tapetum layer began to disintegrate and pollen grains were round. **(I)** SEM of stage 12 in the male flower: the pollen grains were mature and released from the stomium. **(J–N)** Stages 8–12 of female floral development in which detailed description of each stage seen in Figures 4B–F, respectively. br, bract; se, sepal; p, petal; s, stamen; lo, locule; ta, tapetum; te, tetrad; sto, stomium; pg, pollen grain; st, staminode; g, gynoecium; c, carpel; o, ovule; sty, style; gl, gland. Scale bar = 20 um.

In order to further understand the developmental characteristics of male and female flowers, flower development was divided into 12 stages by key morphological events and developmental order of flower organs with the results of SEM and histological section analyses (Figures 2–**5**).

During the vegetative phase of *V. fordii*, the shoot apical meristem (SAM) produced leaves on its flanks. Once transitioned to the reproductive stage, the SAM developed into an inflorescence shoot apical meristem (IM), which was subtended by bracts (stage 1, Figure 2A). A primary IM then further differentiated into floral meristems or secondary inflorescence meristems (stage 2, Figure 2B).

When the IM transformed into a flower primordium, the appearance of the primordia of the sepal, petal, lower layer stamen, and upper layer stamen was defined as stage 3 (Figures 2B,C, **5A**), stage 4 (Figures 2D, **5B**), stage 5 (Figures 2E, **5C**), and stage 6 (Figures 2F, **5D**), respectively. Immediately after stage 6, the male and female flowers began to exhibit some developmental differences, particularly morphological changes in the central dome of the flower primordium. For male flowers, the central dome of the floral meristem retained a plane shape, while the two layers of the stamen primordia continued to grow with an oval shape. This period was defined as stage 7 (Figures 3A,B). In contrast, at stage 7 in the female flower, considerable changes occurred in the morphology of the central dome of the floral meristem. The floral meristem began to bulge and grow upward separate from the stamen primordia, from which the gynoecium arose. The gynoecium was now hemispherical (ca. 220 um in diameter) and the inner layer stamens were pushed between the outer layer stamens (Figure 4A).

At stage 8, two layers of the stamen primordia in male flowers were transformed from oval-shaped to tongue-shaped (Figure 3C). The stamen primordia became stalked at the base. The stalk and the wider upper region would eventually give rise to the filament and anther, respectively (Figures 3C, 5E). For the female flowers, the gynoecium primordium was enlarged and formed a flattened and rounded protrusion (Figures 4B, 5J). The staminodes were constricted at the periphery of the gynoecium primordium and formed a single ring in the third whorl (Figure 4B). The beginning of stage 9 for the male flowers was defined when the anthers adopted convex protrusions on the inner surface, forming locules (Figures 3D, 5F). For the female flowers, the carpel primordia independently and simultaneously appeared at the edge of the gynoecium (Figures 4C, 5K).

Stage 10 was characterized by the occurrence of meiosis in the male flowers. The anther grew rapidly and the tetrads of microspores were differentiated and surrounded by a single layer of tapetal cells (Figure 5G). For the female flowers, the young pistil presented a five-lobed and scalloped cup and the concavities of the cup were clearly evident, corresponding to early locules (Figure 4D). In each locule, the ovule primordia bulged outward, forming an oval shape (Figures 4D, 5L). The staminodes developed in the same manner as in stage 9 for the male flowers. Growing to stage 11, the tapetum layer began to disintegrate and the pollen grains became rounder in the male flowers. The glands also appeared in the male flowers (Figure 5H). At this stage, the five carpels were proximally fused into a single ovary during enlargement in the female flowers (Figure 4E). The petals increased in size and length and the ovules elongated. However, the staminode growth seemed to be retarded and the total length was ca. 150 um. The glands arose at the axil of the petals (Figure 5M).

At stage 12, the pollen grains were mature, ca. 60 um in diameter, and were released from the stomium in the male flowers (Figure 5I). During this stage in the female flowers, the upper part of each carpel differentiated into a short style and the two-split stigma appeared at the distal revolute part of each style (Figures 4F, 5N). The length of the staminodes (ca. 170–220 um) was similar to that of the male flowers in stage 9 and the staminodes disappeared at a later development stage in the female flowers. Soon afterward, anthesis occurred.

As mentioned above, the development of male and female flowers was identical at the first six stages, when the sepals, petals, and stamen primordia were formed (Figures 2A–F, 5A–D). The developmental divergence between the male and female flowers was observed at stage 7 (Figures 3A, 4A). For the female flowers, the central dome of the flower continued to differentiate, forming the gynoecium. Therefore, the female flowers presented bisexual characteristics. The staminodes were retarded and disappeared during subsequent development (Figures 4D–F, 5J–N). Eventually, the female flowers achieved full transformation from bisexuality to unisexuality. Since female sex organs (carpels) never appeared in male flowers, their developmental processes remained unisexual (Figures 3A–D, 5E–H).

### Development of the androecium in male and female flowers

**Figure 6:**
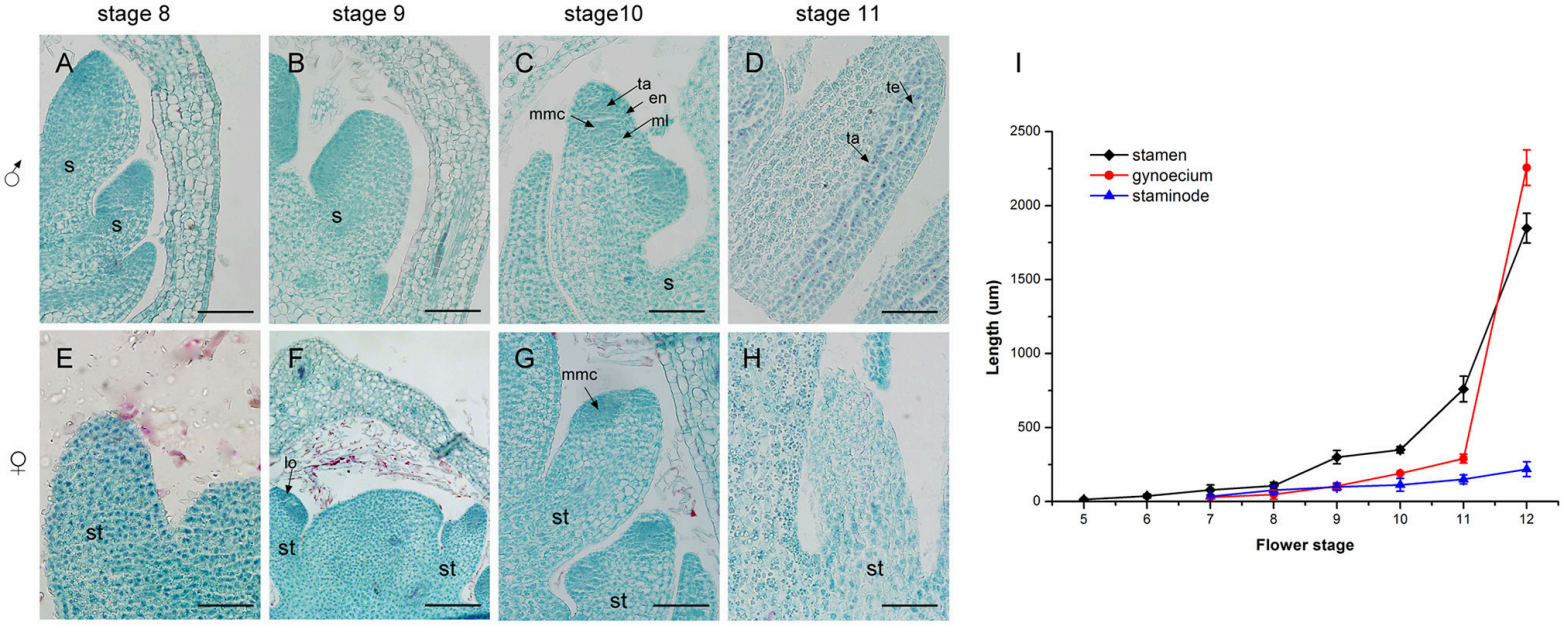
Development of the androecium **(A–H)** and length of flower organs **(I)** in male and female flowers. **(A)** Stamen development at stage 8. **(B)** Appearance of anther and filament at stage 9. **(C)** Meiosis division occurred in locules of the anther at stage 10. **(D)** More pollen grains were surrounded by tapetum at stage 11. **(E,F)** Stage 8 and 9, respectively: the growth of staminode primordium was the same as in stages **(A,B)**. **(G)** At stage 10, staminode development was blocked. **(H)** The staminode cells became vacuolated with sparse cytoplasm density. s, stamen; ta, tapetum; en, endothecium; ml, middle layer; lo, locule; te, tetrad; st, staminode; mmc, microspore mother cell. Scale bar = 100 um.

A comparison of androecium development in male and female flowers was performed, with detailed measurement of the sex organ length in all stages as well as an evaluation of histo-cytological changes in stages 8–11 (Figure 6). At stage 8, the fertile stamen primordia of male flowers was ca. 70 um in length and stamen cells were active with intense stained coloration especially in the nuclei (Figure 6A). At stage 9, the stamens elongated, with differentiated filaments and anthers. During this period, the cell division in the adaxial side of the anther was densely stained and a bilateral structure was established with locules (Figure 6B). At stage 10, the highly stained anthers differentiated and all anther cell types were present including the endothecium, middle layer, tapetum, and microspore mother cells of the locules (Figure 6C). At stage 11, the stamen underwent an increase in length ca. 500 um (Figure 6I) and meiosis occurred in the microspore mother cells, resulting in a tetrad of haploid microspores surrounded by tapetum (Figure 6D).

In contrast to the fertile stamens in male flowers, the staminodes in female flowers presented retarded growth. At stage 8 and stage 9, the staminode cells were densely stained and the anthers of the staminodes were differentiated. This was similar to the stamen development in male flowers at the same stage (Figures 6E,F). However, compared with stamen development in male flowers, the staminodes displayed inconspicuous elongation and their development appeared to stop in stage 10 (Figure 6I). Meanwhile, the anther differentiated abnormally and microspore mother cells could not undergo meiosis to form microspores because of the disappearance of the tetrads in later development stages (Figure 6G). During subsequent development, the staminode cells became vacuolated, the cell differentiation decreased, and the cytoplasm density gradually became sparse from the proximal to the distal region (Figure 6H). In summary, the staminode development was blocked during the development of the anther in stage 10 in the female flower, which led to the transformation of bisexual to unisexual characteristics in female flowers.

### Cell death during staminode abortion

**Figure 7:**
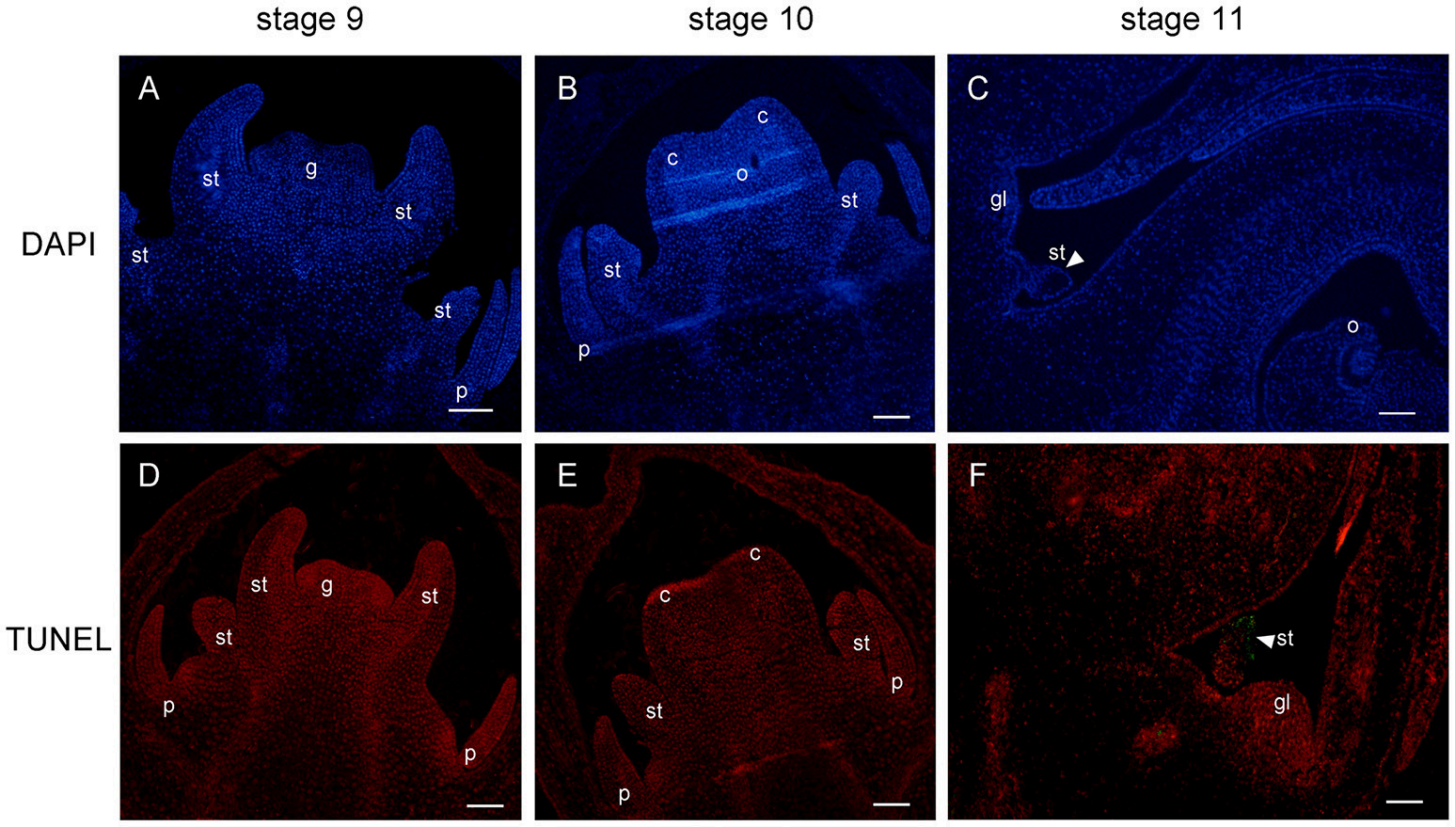
DAPI staining and TUNEL assay of female flower at stages 9, 10, and 11. Staminode showed intact nuclei with bright DAPI at stages 9 **(A)** and 10 **(B)**. At stage 11, nuclear loss (arrowhead) was displayed in subepidermal staminode cells **(C)**. TUNEL signals were not detected at stages 9 **(D)** and 10 **(E)**, and were concentrated (arrowhead) in the staminode at stage 11 **(F)**. st, staminode; g, gynoecium; c, carpel; o, ovule; gl, gland. Scale bar = 100 um.

In order to investigate the mechanism of androecium abortion, we mainly focused on staminode development in female flowers at the last three stages (stages 9, 10, and 11). The cellular changes in aborted staminodes were examined by staining paraffin-embedded tissue sections with DAPI (Figure 7). Figures 7A,B show that there were intact nuclei with bright DAPI staining from staminodes in the female flowers at stages 9 and 10. As shown in Figure 7B, the anther of the staminode underwent differentiation to form a microspore mother cell of the locule with brighter and denser DAPI staining. As the female flowers became mature, the staminodes presented nuclear loss in which diminished DAPI fluorescence was noted in subepidermal cells of the anther at stage 11 (Figure 7C).

In parallel, TUNEL was performed to detect signs of fragmented DNA during the process of staminode abortion. At stages 9 and 10, no TUNEL signal was detected in the staminodes or in the other floral organs (Figures 7D,E). Moreover, the distal anther cells were densely stained by PI, which was consistent with the results in Figure 7B. The TUNEL signals were clearly observed in the subepidermal cells of the anther in the staminode, indicating extensive DNA fragmentation with cell death (Figure 7F). The lightness and density of the proximal-to-distal staminode nuclei gradually declined with DAPI and PI staining, indicating that the loss of nuclear integrity had increased. In brief, the staminode abortion process was determined by cell death with a loss of nuclear integrity and DNA fragmentation in stage 11 during female flower development.

### Illumina sequencing and annotation

In order to investigate the molecular mechanisms responsible for the formation of unisexual flowers in *V. fordii*, the divergence of male and female flowers at stage 7 was sequenced with three biological replicates. In total, 146 million clean reads were generated and each sample produced 16–36 million reads. A total of 56,065 unigenes was assembled with an average length of 1,044 bp and N50 of 1,463 bp. The number of unigenes with over 500 bp was 86.46% (Figure S1).

These unigenes were annotated against the *Arabidopsis* protein database (TAIR 10) and 24,567 (43.82%) were matched. Based on the GO annotations, 21,806 (88.76%) unigenes were assigned to three main categories (cellular component, molecular function, and biological process) and categorized into 29 sub-groups, including “response to abiotic or biotic stimulus,” “response to stress,” “signal transduction,” “developmental processes,” and “transport” (Figure S2). Meanwhile, 8,638 (35.16%) annotated unigenes were subjected to KEGG pathway analysis. They were found to be predominantly involved in the metabolic pathway, biosynthesis of secondary metabolites, ubiquitin-mediated proteolysis, plant hormone signal transduction, and the cell cycle (Figure S3).

### Analysis of differentially expressed genes

According to DEG analysis, 812 DEGs existed between males and females, 608 of which homologously corresponded to unique AGIs (Table S2). A total of 310 and 298 DEGs showed significant expression in male and female flowers, respectively. To validate the DEG expression patterns, 10 transcripts were selected for qRT-PCR (Figure S4). The results showed consistent expression in the qRT-PCR and DEG data.

GO enrichment analysis was performed to reveal significant GO terms classified under three categories (Table 1, Table S3). In the biological process, “Carbohydrate metabolic process” and “Catabolic process” were enriched in both male and female flowers. More GO terms were exclusively enriched in females, including “Response to hormone,” “Response to jasmonic acid,” “Jasmonic acid biosynthetic process,” “Epidermal cell fate specification,” and “Strigolactone biosynthetic process.” Regarding molecular function, “Oxidoreductase activity” and “Hydrolase activity” appeared in the two sexes, while “Polygalacturonase activity” was enriched in males and “Transcription factor activity” was significantly abundant in females. The enriched GO terms for cellular components in males and females were “Vacuole” and “Spliceosomal complex,” respectively. In addition, an overview MapMan analysis showed that most of the different sexual genes were involved in hormone metabolism, development, stress, cell wall, transport, stress, and signaling (Table S4). In the hormone category, jasmonic acid (JA) synthesis genes were significantly expressed in female flowers, which was consistent with the GO enrichment analysis. Interestingly, the genes for photosynthesis, the Calvin cycle, minor CHO metabolism, and glycolysis related genes were specifically expressed in male flowers, which may facilitate stamen development.

**Table 1:** GO ontology (GO) enrichment analysis of DEGs between male and female flowers.

**GO term**	**Ontology**	**Description**	**Male**			**Female**		
			**No**.	***P*-value**	**FDR**	**No**.	***P*-value**	**FDR**
GO:0005975	P	Carbohydrate metabolic process	59	0.000000	0.000000	35	0.000050	0.001185
GO:0009607	P	Response to biotic stimulus	18	–	–	42	0.000000	0.000019
GO:0009056	P	Catabolic process	45	0.000002	0.000153	38	0.000183	0.003038
GO:0009725	P	Response to hormone	28	–	–	38	0.000328	0.004631
GO:0009753	P	Response to jasmonic acid	3	–	–	10	0.002011	0.019786
GO:0009695	P	Jasmonic acid biosynthetic process	–	–	–	4	0.002427	0.023024
GO:0009694	P	Jasmonic acid biosynthetic process	1	–	–	4	0.004073	0.035357
GO:0007568	P	Aging	6	0.049887	–	8	0.003845	0.034045
GO:0010160	P	Formation of organ boundary	–	–	–	3	0.001099	0.012160
GO:0006952	P	Defense response	17	–	–	39	0.000963	0.011125
GO:0009957	P	Epidermal cell fate specification	–	–	–	3	0.000282	0.004109
GO:0042221	P	Response to chemical	53	0.021312	–	58	0.000698	0.008823
GO:1901601	P	Strigolactone biosynthetic process	–	–	–	3	0.000007	0.000261
GO:0006397	P	mRNA processing	–	–	–	13	0.001069	0.012160
GO:0016491	F	Oxidoreductase activity	38	0.002021	0.020992	39	0.000603	0.009313
GO:0016787	F	Hydrolase activity	79	0.000068	0.001726	81	0.000006	0.000237
GO:0004650	F	Polygalacturonase activity	12	0.000000	0.000000	–	–	–
GO:0001071	F	Nucleic acid binding transcription factor activity	8	–	–	28	0.006080	0.047786
GO:0005576	C	Extracellular region	107	0.000000	0.000000	68	0.000000	0.000000
GO:0071944	C	Cell periphery	104	0.000001	0.000035	82	0.017700	–
GO:0005773	C	Vacuole	30	0.001278	0.017167	14	–	–
GO:0005681	C	Spliceosomal complex	–	–	–	7	0.000475	0.006126

*P, biological processes; F, molecular function; C, cellular component*.

### Sexual genes related to phytohormone, transcription factors, and meristem in females

Based on GO enrichment and MapMan analyses, we identified 66 DEGs involved in “Response to hormones,” among which 38 were significantly enriched in female flowers (Table 1, Table S5). These genes that were up-regulated in female flowers encode the *ATP binding cassette subfamily B19, HVA22C, GA3ox2, PIN-LIKE3*, and *LOX2*. These genes are involved in plant organ growth, abscisic acid (ABA) response, gibberellin degradation, auxin transport, and JA synthesis. Meanwhile, the GO terms for “Response to jasmonic acid,” “Jasmonic acid biosynthetic process,” and “Jasmonic acid metabolic process” were also significantly enriched in females (Table 1, Table S6). *Lipoxygenase 2* (*LOX2*) and *Lipoxygenase 3* (*LOX3*) in JA synthesis were up-regulated in females. Moreover, some genes, including *GL3, MYB73*, and *PDR12*, respond to JA signaling, indicating that JA plays vital roles in female flower development.

In this study, 28 genes related to transcription factors (TFs) were significantly expressed in females (Table S7). These TFs were nucleic acid binding transcription factors, including basic helix-loop-helix (bHLH) family proteins (*BR enhanced expression 1, BHLH96*, and *GL3*), MYB domain proteins (*MYB3* and *MYB73*), WRKY DNA-binding proteins (*WRKY33, WRKY46, WRKY53*, and *WRKY60*) and other genes that respond to JA signaling and carpel development. However, eight TFs highly expressed in males were *AP3, Late elongated hypocotyl 1* (*LHY1*), *WLIM1, NAC025*, and *ATRL6*, which predominantly participate in stamen and pollen development.

Compared with male flowers at stage 7, morphological divergence in females consisted of the continuous differentiation of the central dome into the gynoecium, which meant that some genes associated with the floral meristem were specifically expressed in females (Table S8). The four genes *Chromatin remodeling complex subunit R 3* (*CHR3/SYD*), *Argonaute 10* (*AGO10*), *PRP39*, and *Enhanced downy mildew 2* (*EDM2*) are involved in the GO terms of organ boundary specification between lateral organs and the meristem, regulation of timing in meristematic phase transition, regulation of meristem development, and embryonic meristem initiation.

## Discussion

We observed that the differentiation of inflorescences occurred between July and August in the current growing season in Hefei, which is different from the period between May 10 and October 1 reported in previous studies in Florida (Abbott, 1929). The variation in differentiation time may depend on the nutrition level, cultivation, and environmental conditions. For example, high soil moisture can delay the differentiation of inflorescences (Abbott, 1929). The development of inflorescences started from July and lasted until April of the following year (Figure 1). Similarly, this long period of inflorescence bud development can be seen in many species, such as pistachio (*Pistacia vera*), Chinese ixora (*Ixora chinensis*), and apples (*Malus domestica*) (Golan-Goldhirsh et al., 1998; Chen et al., 2003; Foster et al., 2003). Although both *V. fordii* and *Jatropha curcas* belong to the Euphorbiaceae family, the period of inflorescence differentiation for *J. curcas* is ~1 month (Alam et al., 2011), indicating that each species has its own pattern for inflorescence differentiation. *V. fordii* inflorescences are cymose panicles that branch dichotomously or trichotomously on the terminal shoot, showing a type of definite inflorescence. Typically, female flowers are located at the center of the main or secondary axes, which are closed in many male flowers. This facilitates self-pollination and the generation of homozygous progeny for several successive generations, which could further assist cultivar improvement and genetic study (Potter, 1959).

Histological and SEM analyses showed that the first divergence of sexual differences emerged at stage 7 and from Figures 3A, 4A, we could demonstrate that the meristematic size in females (ca. 200 um in length) is larger than that of in male flowers (ca. 70 um in length). We postulated that some genes could directly regulate proper flower development and meristematic activity. MADS-box TFs are required for floral organ identity specification (Krizek and Fletcher, 2005). According to the classical ABC model, class B, and class C genes collectively define stamens identity; class C genes alone determine formation of carpels (Coen and Meyerowitz, 1991; Weigel and Meyerowitz, 1994). As expected in DEGs libraries, the class B gene (*AP3*) in *V. forii* was significantly increased in male flowers, which was consistent with the more expresson of *S. latifolia SLM3* (an *AP3* homolog) in male flowers than female flowers (Lebel-Hardenack and Grant, 1997). And the class C gene (*Agamous*) in *V. fordii* has similar expression in both male and female flowers. The SAM niche comprises undifferentiated and dividing stem cells that maintain the plant meristem and can differentiate to form new organs (Galli and Gallavotti, 2016). Combined with transcriptomic data, two genes (*SYD* and *AGO10*) related to SAM fate, were up-regulated in female flowers. *SYD*, which encodes a SWI2/SNF2 ATPase in the SNF2 subclass, regulates the SAM identity with *LEAFY* as well as the development of the proper carpel and ovule (Wagner and Meyerowitz, 2002). *SYD* is recruited to the *WUSCHEL* promoter and is involved in *CLAVATAL-WUSCHEL* feedback loop signaling, which coordinates SAM cell maintenance, differentiation, and proliferation (Kwon et al., 2005; Somssich et al., 2016). There is another signaling pathway in which the SAM is positively regulated by *AGO10*, along with *WUSCHEL* and *CLAVATA* genes. AGO10 maintains stem cell homeostasis in SAM and specifically binds to miR166/165, repressing its incorporation into AGO1 complexes (Zhu et al., 2011; Yu et al., 2017). Therefore, the meristem-related genes *SYD* and *AGO10* could be considered as candidate regulators of sex determination in *V. fordii*.

Next, we focused on the exact morphological and cellular changes in the staminode of female flowers with a detailed histological analysis (Figure 6). The data indicated that at an early stage, the staminode continued to differentiate into the anthers with a bilobal stage and the development of short, non-elongated filaments. In stage 10, the sporogenous cell underwent mitosis, forming microspore mother cells with great bulk, large nuclei, and dense cytoplasm. Subsequently, the anthers degenerated and the microspore mother cells did not differentiate into microspores through meiosis at the floral stage 10, which was recognized as the pre-meiosis stage. Similarly, the arrest of the staminodes can occur at any stage of development in other diclinous species, such as, at the initiation of the stamen primordia in *P. dactylifera* (Daher et al., 2010), at the early stages of anther primordia development in *C. sativus* (Hao et al., 2003), with the development of sterile microspores in *V. vinifera* (Caporali et al., 2003), and at the early sporogenous stage in *S. latifolia* (Farbos et al., 1997). There are two predominant developmental processes for inappropriate sex organ arrest in unisexual flowers: cell cycle block and cell death (Diggle et al., 2011). Developmental studies revealed that the arrest of staminodes in *V. fordii* female flowers resulted from cell death, as detected by DAPI and TUNEL assays, at stage 11 of flower development (Figure 7). In maize, pistil abortion in the tassel and secondary ear is associated with cell death mediated by *Tasselseed1* and *tasselseed2*, whereas stamen arrest in ear spikelets is involved in cell cycle block (Calderon-Urrea and Dellaporta, 1999; Kim et al., 2007). In *P. dactylifera*, the abortion of sterile sex organs (staminodes and pistillodes) is related to cell cycle arrest with nuclear integrity (Daher et al., 2010). Thus, a great variety of developmental arrest stages and processes suggest that each species has independent genetic mechanisms that control inappropriate sex organs under unisexual flower development. However, the genetic mechanisms underlying the developmental abortion of the staminode in *V. fordii* remain to be determined.

Phytohormones play important roles in flower development, especially in sex determination (Tanurdzic and Banks, 2004). Comparative transcriptome analysis showed that the expression of some genes involved in auxin, cytokinin, ethylene, gibberellin, and ABA biosynthesis was significantly changed. However, GO term enrichment demonstrated that some DEGs involved in JA biosynthesis were significantly enriched in female flowers, suggesting that JA could play crucial roles in the sex determination process in *V. fordii*. In *Arabidopsis, LOX2* is highly expressed in inflorescences and contributes to the majority of jasmonate synthesis (Bell and Mullet, 1993). *Lox3 lox4* double mutants display more inflorescence shoots and flowers with carpelloid and staminode structures (Caldelari et al., 2011), which suggests that JA is related to the termination of meristematic activity and affects sex determination. In *V. fordii*, the homologs of these genes are all up-regulated in female flowers, implying that JA biosynthesis is higher in females than in males and JA plays essential roles in floral meristem activities that lead to the differentiation of gynoecium. As in tomato (*Lycopersicon esculentum*), *jasmonic acid*-*insensitive 1* (*jai1*) mutants defective in JA signaling exhibit a defect in the maternal process of seed production, which indicates that JA also plays an important role in female fertility (Li et al., 2004). However, JA is a positive regulator in the stamen development of the tassels in maize that alter sex determination. *Tasselseed1* mutants display pistil development of the primary and secondary florets in both ears as well as tassels with no stamen development and stamen development can be rescued by the application of exogenous JA (Irish et al., 1994; Acosta et al., 2009). The roles of JA in maize, *Arabidopsis* and tomato demonstrate that JA regulates distinct developmental processes in different species. We propose that JA triggers tissue- or cell-specific signaling to reprogram the differentiation of the gynoecium for the sexual dimorphism of female flowers in *V. fordii*. Meanwhile, transcriptomic analysis showed that some TF families response to JA signaling, including bHLH, WKRY and MYB, were identified in the DEGs between male and female flowers. *GLABRA3* (*GL3*) belongs to the bHLH TF family. *GL3* was up-regulated in female flowers and is involved in the JA response and cell fate (Yoshida et al., 2009). *MYB3* and *MYB73* were also up-regulated in female flowers. *MYB3* belongs to subgroup 4 of the R2R3-type MYB family (Stracke et al., 2001) and is strongly induced in stamens by JA treatment in *Arabidopsis* (Mandaokar et al., 2006), which is consistent with the higher JA concentration that leads to sexual dimorphism in *V. fordii. MYB73* belongs to subgroup 22 of the R2R3-type MYB family (Stracke et al., 2001) and the expression of *MYB73* is significantly decreased in *jasmonate resistant 1(jar1)* mutants, indicating that *MYB73* is involved in JA signaling (Jiao et al., 2011). These results suggest that female TFs are associated with JA that modulates sex determination in *V. fordii*.

In conclusion, the present study provides a detailed description of *V. fordii* floral morphogenesis and the formation of unisexual flowers. Based on transcriptomic analysis of male and female flowers in *V. fordii*, we can preliminarily understand the regulatory networks involved in sexual dimorphism. Some genes associated with the JA biosynthesis pathway and JA metabolic processes were significantly modified in female flowers. Additionally, a number of the TFs responded to JA signaling and triggered extensive transcriptional reprogramming on the floral meristem in *V. fordii*. These data provide deeper insights to study the processes of sex determination in *V. fordii*.

## Author contributions

YM and LW conceived and designed the experiments. YM and WBL performed the histological analysis. YM, XC, and YX performed the scanning electron microscopy. YM analyzed sequencing data. YM and LW wrote the manuscript and WLL, JH, JN, YW reviewed the manuscript.

### Conflict of interest statement

The authors declare that the research was conducted in the absence of any commercial or financial relationships that could be construed as a potential conflict of interest.

## Supplementary material

Figure S1 The distribution and proportion of all assembled contig lengths.

Figure S2 Gene ontology (GO) classification for all assembled unigenes using SuperViewer tool with normalized results.

Figure S3 KEGG pathway analysis for DEGs.

Figure S4 qRT-PCR validation of DEGs data in *V. fordii*.

Table S1 Primers used for validation of DEGs in qRT-PCR.

Table S2 The 812 genes differentially expressed between male and female flowers in stage 7.

Table S3 GO term enrichment analyses of male (left) and female (right) flowers in *V. fordii*.

Table S4 Functional categories of differentially expressed genes assigned by MapMan.

Table S5 Genes enriched to GO term “response to hormone” in female flowers.

Table S6 Genes enriched to GO term related to JA in female flowers.

Table S7 Genes related to transcription factors in male and female flowers.

Table S8 Genes related to floral meristem in female flowers.
