# Supplementary figures and images for: Flower Development and Sex Determination between Male and Female Flowers in Vernicia fordii
Source: Front Plant Sci. 2017 Jul 20;8:1291. doi: 10.3389/fpls.2017.01291 (PMC5517574; doi:10.3389/fpls.2017.01291)

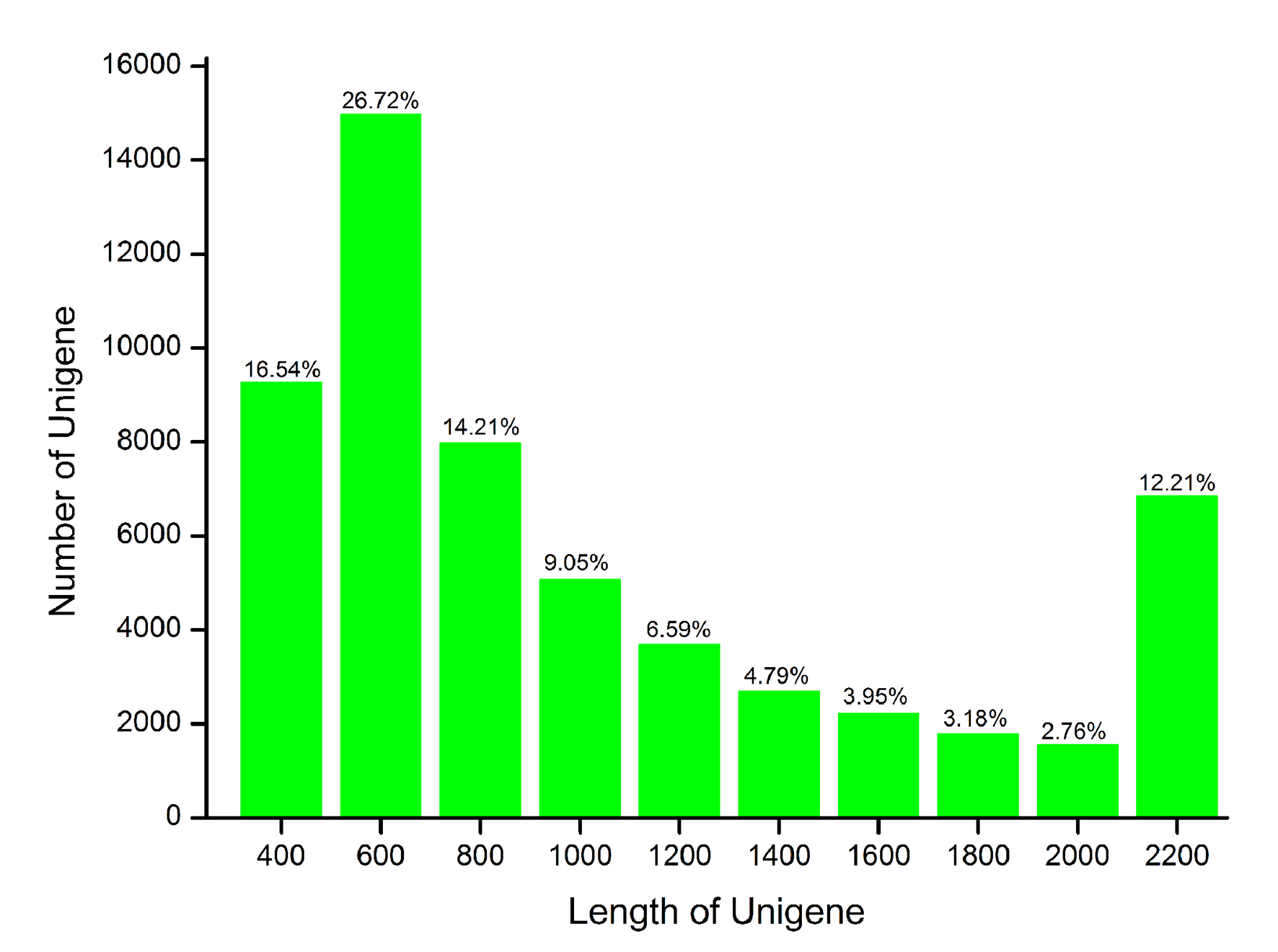

Supplement: Figure S1 — The distribution and proportion of all assembled contig lengths. [file Image1.TIF]

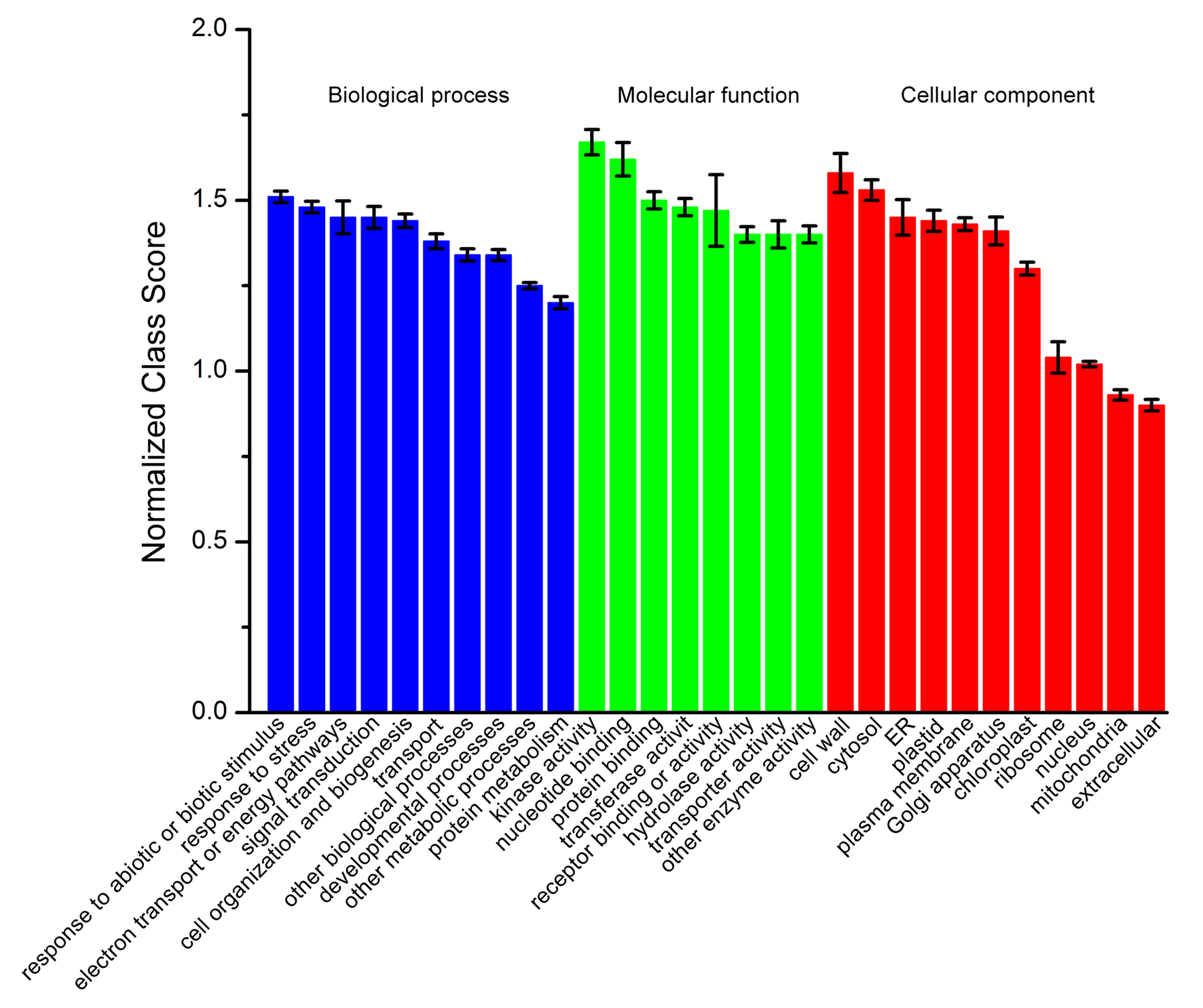

Supplement: Figure S2 — Gene ontology (GO) classification for all assembled unigenes using SuperViewer tool with normalized results. [file Image2.TIF]

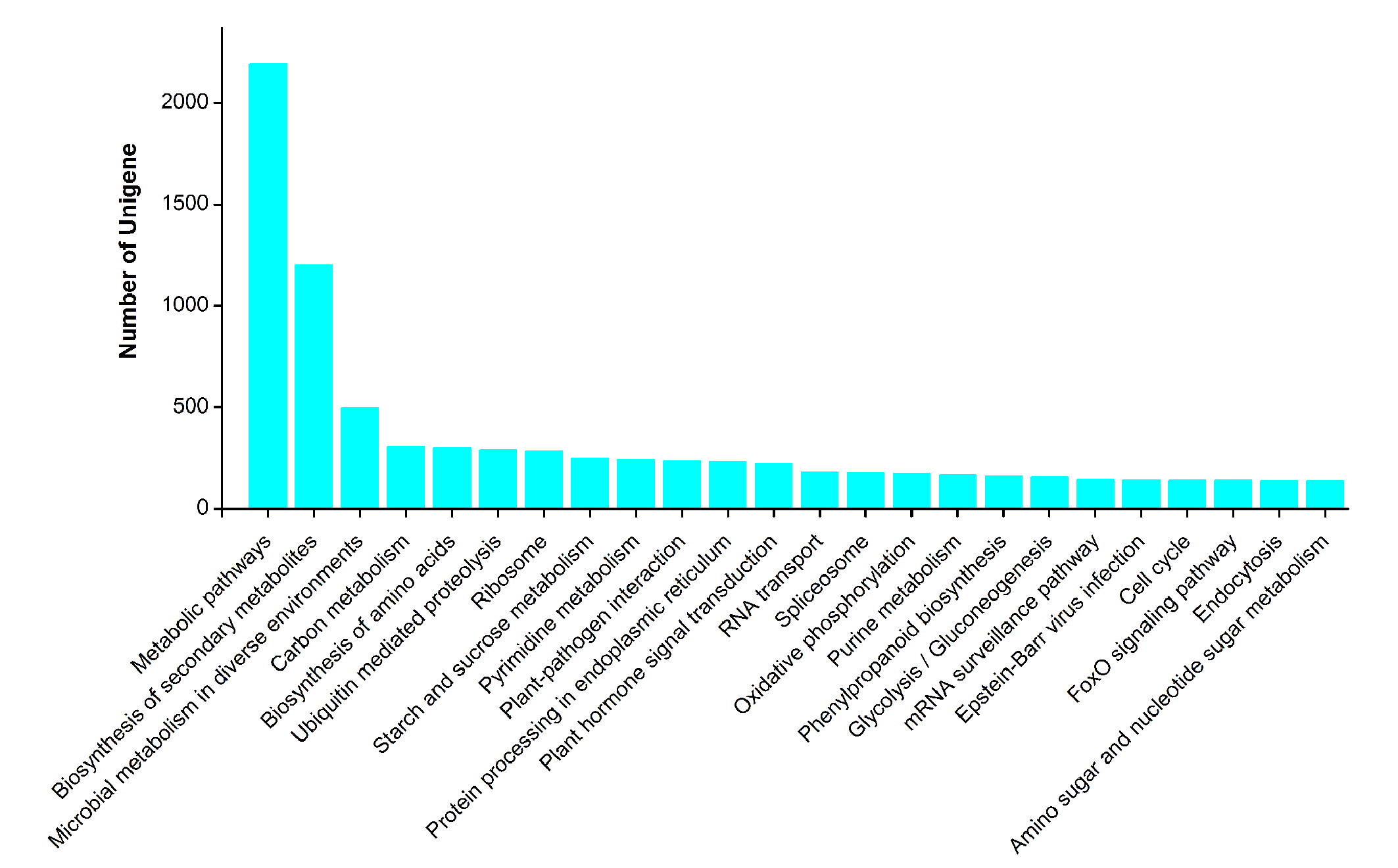

Supplement: Figure S3 — KEGG pathway analysis for DEGs. [file Image3.TIF]

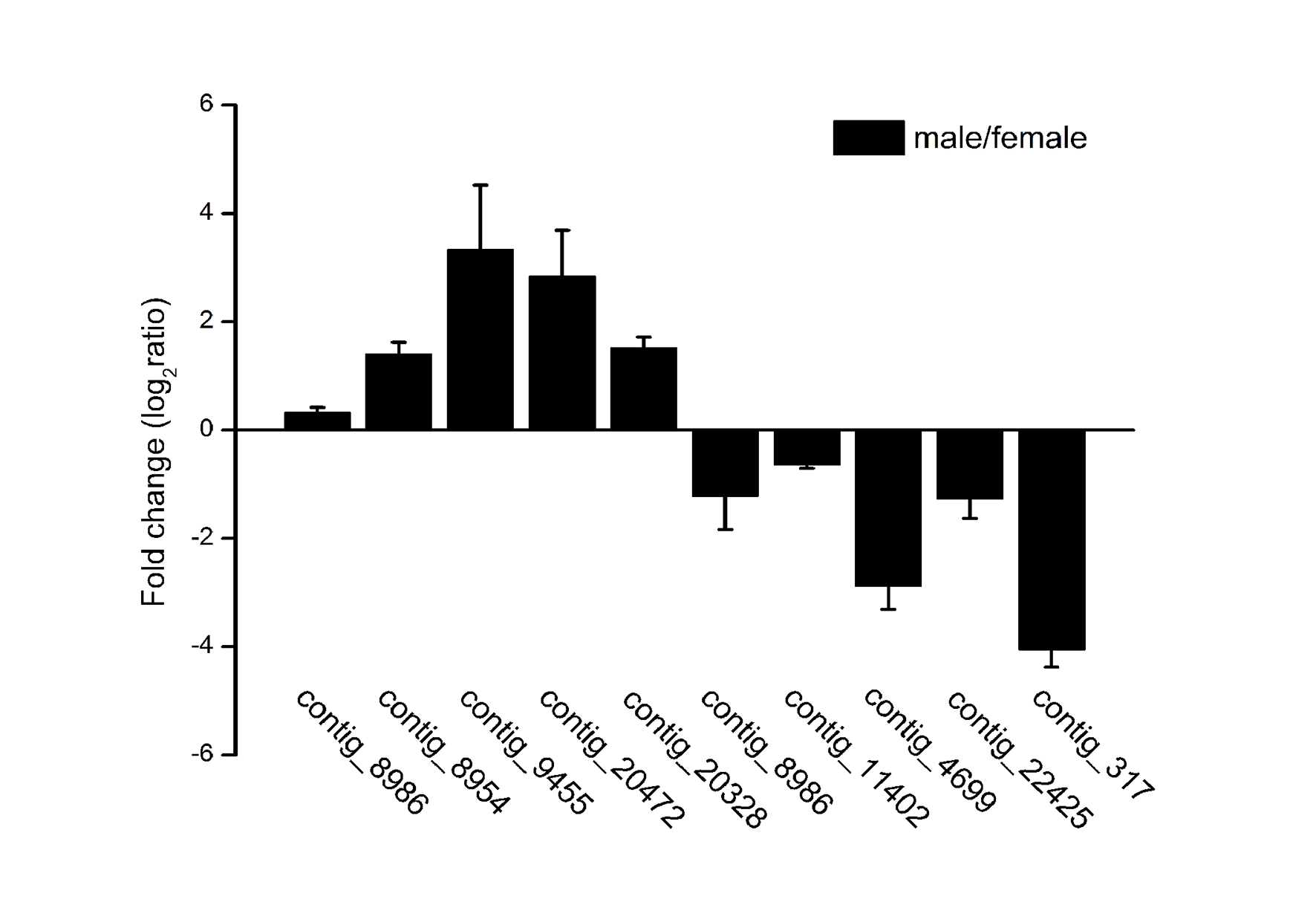

Supplement: Figure S4 — qRT-PCR validation of DEGs data in V. fordii. [file Image4.TIF]
